# Techniques and Current Role of Sentinel Lymph Node (SLN) Concept in Gastric Cancer Surgery

**DOI:** 10.3389/fsurg.2018.00077

**Published:** 2019-01-22

**Authors:** Dimitrios Symeonidis, Konstantinos Tepetes

**Affiliations:** Department of Surgery, University Hospital of Larissa, Larissa, Greece

**Keywords:** sentinel node, gastric cancer, lymphadenectomy, gastrectomy, laparoscopic assisted gastrectomy

## Abstract

Gastric cancer patients represent a rather divergent patient group and in certain carefully selected cases of early forms of gastric cancer the D2 gastrectomy could be considered a more radical procedure than the biological and oncological characteristics of the primary tumor on the gastric wall would require. As any unnecessary dissection increases morbidity without always respective survival benefits, an approach that could accurately predict and actually dictate the exact extent of lymph node dissection would be ideal. It is more than logical the assumption that the standard D2 lymphadenectomy could represent an overtreatment in distinct patients groups such as patients with early gastric cancer with favorable pathological characteristics and clinically negative nodes not suitable for endoscopic treatment because this early stage disease shows limited lymph node metastasis incidence and excellent overall survival. Considering that the D2 gastrectomy has a negative impact on the quality of life of gastric cancer patients due to the post-gastrectomy functional results, a concept of a more targeted lymph node dissection, when appropriate, is certainly appealing. It is yet to be proven whether sentinel lymph node navigation surgery can fulfill such expectations providing the appropriate balance between morbidity and oncological safety in selected gastric cancer patients.

## Introduction

Gastric cancer is a rather common malignancy, the 6th most common according to the GLOBOCAN 2012 data, with a reported mortality of 8.9/100,000 population (1). Although a multimodality treatment approach is warrant, surgery justifiably remains the cornerstone of treatment. Though the complete surgical excision of the primary tumor on the gastric wall is common place, there has been an extensive debate in the literature in regards to the most proper lymph node dissection extent. Traditionally, Eastern Asian surgeons stated in favor of the extended (D2) lymphadenectomy due to the anticipated better loco-regional control of the disease. On the other hand, the increased morbidity and mortality attributed initially to the D2 lymphadenectomy by the three major trials on the field, the Medical Research Council (MRC), the Dutch and the Italian randomized control trial, without respective survival benefits had led Western surgeons toward a more limited lymphadenectomy i.e., the D1 (2–4). However, the long term (15 year) follow up of the Dutch trial changed the general picture as the authors reported a significant decrease in recurrence rate after D2 procedure and attributed the D2 associated morbidity and mortality to the spleno-pancreatectomy that was routinely performed in the D2 patient group. D2 lympadenectomy is now suggested as the recommended procedure for all patients with resectable advanced gastric cancer (5).

As the D2 gastrectomy is a procedure involving major dissections, a quite high associated morbidity and mortality should be anticipated even when performed in highly specialized centers (5). Adapting the methodological principles currently followed and became well-established in the surgical treatment of other malignancies such as melanoma, breast and thyroid cancer, the concept of sentinel node mapping, and biopsy has been proposed in the gastric cancer surgical treatment as well (6, 7). The actual goal behind this approach was to pass on the already well-established advantages of limited surgery i.e., less associated morbidity and mortality to a, by definition highly morbid patient population, such as the patients operated upon gastric malignancies.

Gastric cancer patients represent a rather divergent patient group and in certain cases such as patients with early gastric cancer with favorable pathological characteristics and clinically negative nodes the D2 gastrectomy could be considered a more radical procedure than the biological and oncological characteristics of the primary tumor on the gastric would optimally require. In support of the above, the Japanese Gastric Cancer Association guidelines currently advice a more limited either a D1 or a D1+ lymphadenectomy in these favorable early forms of gastric cancer (8). Considering that the D2 gastrectomy significantly decreases the quality of life of patients due to the post-gastrectomy functional results, a concept of a more targeted and focused lymph node dissection appears especially attractive. It is this certain field where the sentinel lymph node navigation surgery is aiming to provide the appropriate answers and actually become the new paradigm in EGC treatment (2).

The sentinel node concept is based on the main principle that the status of the sentinel node i.e., whether it is infiltrated by metastasized malignant cells or not can predict and finally reflect the status of the remaining nodes as well. Under this prism, a negative for metastasis sentinel node could indicate that no other lymph node is infiltrated by malignant cells. Taking this concept one step further, a properly standardized sentinel lymph node navigation surgery could ideally create the conditions for a precise and consequently limited lymph node dissection probably required in selected patient group. On top of all, as the penetration of laparoscopic surgery in gastric cancer treatment armamentarium has recently increased, the surgical community's interest on the topic has been triggered. In the present review, we aimed to provide comprehensive and updated answers to simple questions regarding the technical aspects, the validity of the technique itself as well as the certain indications of the sentinel lymph node navigation concept in gastric cancer surgery.

## Gastric Lymph Node Stations

Traditionally, the importance of the lymph node status in gastric cancer was widely acknowledged and appreciated especially by Eastern Asian surgeons. In line with this concept, back in 1973 the Japanese Research Society for the study of gastric cancer aimed to define the lymphatic drainage pattern of the stomach and subsequently standardize lymph node dissections (9). This initial classification has been thoroughly revised until the latest Japanese Gastric Cancer Association classification published on 2011 where a detailed description of the regional lymph nodes of the stomach is provided (8) (Table 1).

**Table 1:** Anatomical definitions of lymph node stations (LNs, Lymph Nodes).

**No**.	**Definition**
1	Right paracardial, including those along the first branch of the ascending limb of the left gastric artery.
2	Left paracardial, including those along the esophagocardiac branch of the left subphrenic artery
3a	Lesser curvature LNs along the branches of the left gastric artery
3b	Lesser curvature LNs along the 2nd branch and distal part of the right gastric artery
4sa	Left greater curvature LNs along the short gastric arteries (perigastric area)
4sb	Left greater curvature LNs along the left gastroepiploic artery (perigastric area)
4d	Rt. greater curvature LNs along the 2nd branch and distal part of the right gastroepiploic artery
5	Suprapyloric LNs along the 1st branch and proximal part of the right gastric artery
6	Infrapyloric LNs along the first branch and proximal part of the right gastroepiploic artery down to the confluence of the right gastroepiploic vein and the anterior superior pancreatoduodenal vein
7	Along the trunk of left gastric artery between its root and the origin of its ascending branch
8a	Anterosuperior LNs along the common hepatic artery
8p	Posterior LNs along the common hepatic artery
9	Celiac artery LNs
10	Splenic hilar LNs including those adjacent to the splenic artery distal to the pancreatic tail, and those on the roots of the short gastric arteries and those along the left gastroepiploic artery proximal to its 1st gastric branch
11p	Proximal splenic artery LNs from its origin to halfway between its origin and the pancreatic tail end
11d	Distal splenic artery LNs from halfway between its origin and the pancreatic tail end to the end of the pancreatic tail
12a	Hepatoduodenal ligament LNs along the proper hepatic artery, in the caudal half between the confluence of the right and left hepatic ducts and the upper border of the pancreas
12b	Hepatoduodenal ligament LNs along the bile duct, in the caudal half between the confluence of the right and left hepatic ducts and the upper border of the pancreas
12p	Hepatoduodenal ligament LNs along the portal vein in the caudal half between the confluence of the right and left hepatic ducts and the upper border of the pancreas
13	LNs on the posterior surface of the pancreatic head cranial to the duodenal papilla
14v	LNs along the superior mesenteric vein
15	LNs along the middle colic vessels
16a1	Paraaortic LNs in the diaphragmatic aortic hiatus
16a2	Paraaortic LNs between the upper margin of the origin of the celiac artery and the lower border of the left renal vein
16b1	Paraaortic LNs between the lower border of the left renal vein and the upper border of the origin of the inferior mesenteric artery
16b2	Paraaortic LNs between the upper border of the origin of the inferior mesenteric artery and the aortic bifurcation
17	LNs on the anterior surface of the pancreatic head beneath the pancreatic sheath
18	LNs along the inferior border of the pancreatic body
19	Infradiaphragmatic LNs predominantly along the subphrenic artery
20	Paraesophageal LNs in the diaphragmatic esophageal hiatus
110	Paraesophageal LNs in the lower thorax
111	Supradiaphragmatic LNs separate from the esophagus
112	Posterior mediastinal LNs separate from the esophagus and the esophageal hiatus

According to this classification, the lymph originating from the stomach is drained via lymphatics and in turn is filtered through lymph nodes which are classified into distinct stations numbered from 1 to 20 plus stations 110, 111, and 112. Lymph node stations 1–12 as well as station 14v represent the regional stations while the rest of the lymph node stations represent the distant ones. Lymph node stations No. 19, 20, 110, and 111 represent regional lymph nodes in case of tumors invading the esophagus (8).

## Lymphatic Stream in Gastric Cancer

It becomes obvious that the stomach has a pretty complex lymphatic drainage pattern and a thorough knowledge of this pattern is of paramount importance especially for surgeons dealing with gastric malignancies (10). Nevertheless, this is not always enough because tumors of any location within the stomach can metastasize in a non-predictable manner skipping the anatomically anticipated lymph node stations. Several factors have been implicated as contributors for increasing the risk for an atypical metastasis. Tumor location is one of those. In general, tumors located longitudinally at the lower part of the lesser curvature or circumferentially at the lesser curvature have a higher chance of atypical metastasis compared to other locations (11). A carefully designed approach is warrant when dealing, in the form of sentinel node navigation surgery, with primary tumors at these locations within the stomach in order to prevent false negative results. A skip metastasis incidence of up to 29% has been reported in these cases (11).

In addition, the degree of tumor differentiation has been studied and analyzed in this direction as well. Poorly differentiated tumors seem to metastasize in an out of the ordinary manner more often and labeling such tumors as high risk for a skip metastasis is a justifiable approach (12). In general, the severity of gastric malignancy determined by the size of the primary tumor and/or the depth of invasion, the T stage of the TNM classification, is directly related with the lymph node metastasis rate (13). In regards to the topography of the involved lymph nodes, a retrospective study which compared patients with solitary lymph node involvement with patients without a lymph node metastasis demonstrated that the majority of sentinel lymph nodes are located in the regional peri-gastric lymph node groups close to the primary tumor (14). Taking this concept one step further, Lee et al. suggested that if sentinel nodes are not found in the usual culprit locations i.e., the peri-gastric lymph nodes, lymph node stations No. 7, 8, and 9 should be additionally explored preventing in this way possible false-negative sentinel node mapping results (15).

## Which Are the Most Commonly Used Tracers for Sentinel Node Mapping?

An ideal tracer is non-toxic, easily available, and cost effective. Optimally, the tracer should accumulate within the lymphatic plexus of the stomach effectively and in a manner that renders its identification possible and optimally without the need of using highly sophisticated equipment. Unfortunately, none of the available substances that have been used as tracers meet the, previously mentioned, ideal requirements rendering the comparison and test of tracers, with different biochemical characteristics within properly designed studies, mandatory. Traditionally, dyes, and radioisotopes represent the two main categories of tracers. Patent blue, lymphazurin, and the indocyanine green are the most commonly used dyes (16). Dye-based methods have achieved high penetration levels among institutions mainly due to cost effectiveness reasons. The additional benefit of detecting both the lymphatic vessels and the lymph nodes as well permits an accurate visualization of the lymphatic drainage pattern out of the malignant site. However, the efficiency of the dye-based mapping technique is compromised in patients with dense adipose tissue decreasing to non-diagnostic the levels of lymph node detection (17). In addition, there are indeed studies reporting often poor visibility with the dye alone technique (18).

In an attempt to overcome this problem, infrared ray electronic endoscopy has been combined with indocyanine green in order to achieve superior visualization results (19). In this technique, a fluorescence imaging system provides real time visualization of the gastric lymphatic tree allowing a pretty accurate mapping of the surgical anatomy (19). This combined technique exhibits superior results (higher sensitivity and higher accuracy) compared to the ICG injection alone illuminating sentinel lymph nodes and lymphatic vessels not visible on the ICG alone technique. The previous disadvantage of poor visualization in the case of patients with dense adipose tissue seems to be adequately addressed by the adjuvant use of infrared ray electronic endoscopy (20).

In regards to the radio-isotopic method, the technetium—99 m tin colloid, the technetium—99 m sulfur colloid, and the technetium—99 m antimony sulfur colloid have all been tested as tracers (16). From the technical viewpoint, a gamma probe is required during surgery to visualize the, previously injected through endoscopy, radioisotope. The use of radioisotopes instead of dyes as tracers appears to have certain advantages such as additional objectivity in the interpretation and the reproduction of the mapping result and the ability to achieve high levels of discrimination even in patients with a dense adipose tissue. Due to the increased retention time of the radioisotope within the labeled lymph node, this latter technique could be optimally combined with laparoscopic surgery. The limitations include the sophisticated equipment required as well as the high cost of the radioactive substance (16).

Trying to appraise the above, it becomes clears that the advantages of the two methods i.e., the dye and the radio isotope based technique are mainly complementary. Thus, the combination of tracers (dye with radioisotope) has become especially popular, an approach aiming to seize and employ the benefits of each tracer at the same time. Indeed, the anticipated increase in the detection rate accuracy by the use of a double tracer has been confirmed by several literature reports and the combined technique has achieved global acceptance among experts (21, 22). However, as the optimal single tracer has not been invented so far, the ongoing research either in the field of tracers or in the visualization systems is imperative. Recently, a hybrid single-photon emission computed tomography/computed tomography (SPECT/CT) system was proposed as capable to provide precise CT images for sentinel node mapping in various types of malignancies such as thyroid cancer (23, 24). In the near future, applications of this technique might become available in gastric cancer navigation surgery as well.

## How is the Tracer Administered?

Two different methods have been tested for the administration of the tracer around the primary tumor. The first method includes the injection of the tracer into the sub-mucosal layer of the gastric wall around the primary tumor under endoscopic examination while a sub-serosal injection during the surgical procedure represents the second option. The dye (usually 2 ml of indocyanine green solution) is injected into the sub-mucosa or the sub-serosa and is distributed evenly around the primary tumor while 2.0 ml of Technetium–99 m colloid solution is injected (usually a day prior to surgery) in a similar manner using endoscopy (25).

In general, studies failed to demonstrate a significant difference between the sub-mucosal and the sub-serosal injection method in regards to the sentinel lymph node identification rates (26). However, the sub-mucosal injection via endoscopy currently represents the mostly utilized method among institutions. The higher penetration of the endoscopic injection among the specialized institution could be explained by the fact that this technique is optimally combined with laparoscopic surgery. From the surgeon's viewpoint, one of the disadvantages of this minimally invasive surgical procedure is the lack of tactile sensation rendering the identification of small tumors during laparoscopy and therefore the subsequent sub—serosal injection often impossible (26).

## Which is the Best Method for Collecting Sentinel Nodes?

In general, labeled lymph nodes by the used tracer either dye or radioisotope or both are collected for further examination. Two distinct methods for this procedure have been described and analyzed in the literature. The picked-up method implies the removal of the labeled nodes only. This is currently the technique used to assess the sentinel lymph node status in breast cancer and melanoma. The other method is the, so called in the literature, lymphatic basin dissection (27). The gastric lymphatic basins represent group of nodes usually distributed along the direction of the main arteries. In real mapping time, the lateral borders of the lymphatic basin are marked by the dyed lymphatic vessels (when the dye based method is used) while the proximal and the distal borders are the stomach wall and the most distal stained node, respectively (28).

Consequently, lymphatic basin dissection is a selective type of lymphadenectomy which includes the en bloc dissection of the basins where all stained with dye lymph nodes are collected and sent out for pathological analysis intra-operatively (29, 30). The accuracy rate of lymph node metastasis in the lymphatic basin dissection method (92.3%) appears superior than the reported accuracy of the pick-up method (50%). Thus, studies suggest that this backup dissection performed during the lymphatic basin dissection could minimize the risk of missed sentinel nodes offering superior oncological results than the ordinary pick up method (31, 32). Certainly, the technique of lymphatic basin dissection as proposed alternative to the ordinary pick-up method is not the answer to all existing limitation of the sentinel lymph node navigation surgery. Further calibration and testing of the technique within properly designed studies is necessary.

## How is a Metastasis in the Retrieved Lymph Nodes Verified?

Probably the most controversial and troublesome issue in the logistics of the sentinel node concept in gastric cancer surgery is the establishment of an accurate intra-operative diagnosis of lymph node metastasis in a timely manner. Usually, the intraoperative diagnosis of a lymph node metastasis is established by examining the frozen section of the stained node by the classic hematoxylin—eosin staining (33). A notable variance in the reported accuracy in the diagnosis utilizing the hematoxylin/eosin staining is obvious in the literature (range 74–100%) raising logical questions regarding the efficiency of this classic staining technique (34). In support of these concerns, the JCOG0302 study of the Japan Clinical Oncology Group (JCOG) was prematurely terminated due to the high false negative rate of the intraoperative pathology performed by the use of hematoxylin/eosin staining. This failure was mainly attributed to the fact that the intraoperative histological examination was conducted by the examination of only a single plane of the retrieved nodes (35).

In an attempt to properly address this issue, molecular techniques have been utilized such as the reverse transcriptase—polymerase chain reaction (RT-PCR) or the one-step nucleic acid amplification assay (32, 36). Arigami et al. compared the efficiency of the different available techniques i.e., hematoxylin/eosin, immunohistochemical staining and RT-PCR for the pathological assessment of the picked up lymph nodes reporting detection rates of 8.2, 13.1, and 36.1%, respectively (37). In general, although there is no clear consensus about the definitive clinical significance of lymph node micro-metastases, the clinical impact of lymph node micro-metastases seems to be indeed remarkable in gastric cancer. For minimally invasive treatments in particular, such as endoscopic sub-mucosal dissection and laparoscopic surgery, the accurate diagnosis of lymph node micro-metastases is regarded as the crucial factor in maintaining the balance between curability and safety (38). Aiming to limit to minimum the false negative cases, the concept of performing a wider but still limited dissection i.e., the sentinel node basin dissention concept sounds at least logical. Kinami et al. did not document any recurrences in 190 patients who were diagnosed as node-negative treated by sentinel node basin biopsy intra-operatively and function-preserving gastrectomy (28).

Similarly, a recent study showed that the use of RT-PCR with the carcino-embryonic antigen as mRNA shows superior sensitivity rates compared with immune-histochemistry for identifying micro-metastasis in the retrieved lymph nodes (35, 37). The authors reported that although the incidence of micro-metastasis detected by RT-PCR was quite high in the given patient sample, sentinel node navigation identified such metastasis in all patients except one who however had a cT2 tumor. Thus, they concluded that the sentinel node concept was applicable to patients with cT1 and cN0 gastric cancer, even when micro-metastasis was detected by the use of RT-PCR (37). Shimizu et al reported that real time multiplex RT-PCR assay for the expression of cytokeratin 19, cytokeratin 20 and CEA is a useful tool for the detection of micro-metastases in sentinel and non-sentinel nodes in gastric cancer patients (39). However, regardless of the actual natural history of gastric cancer micro-metastasis, the universal availability and use of such, highly specialized, detection techniques remains especially doubtful.

## Which Gastric Cancer Patients are Eligible for Sentinel Node Mapping And Biopsy?

Although sentinel node navigation surgery in gastric cancer has several well-documented advantages, there are indeed limitations and drawbacks in the technique as well. Thus, proper patient selection is of paramount importance in order to highlight the benefits of the technique and not violate the basic oncological principles. Literature reports raise the eligibility to as high as 50% (range 3–50%) of gastric cancer patients (31, 40–43). Studies from Eastern Asian institutions have applied the sentinel node navigation surgery to node negative T1 and T2 gastric cancer patients (31, 44–47) while Western studies have tested the concept to gastric cancer patients of more advanced T stage i.e., T3 as well (22). The main problem that could compromise the oncological efficiency of the procedure in these patient groups is skip metastasis. The approach of lymphatic basin dissection could, at least theoretically, address the skip metastasis issue as these metastases tend to be confined in the immediate vicinity of the labeled nodes (48).

## What Are the Surgical Options for the Primary Tumor in Sentinel Node Navigation Surgery?

The advantages of laparoscopic surgery over conventional surgery such as less postoperative pain, decreased length of hospital stay, superior cosmetic results, and earlier recovery are now widely accepted. In gastric cancer surgery however, questions were raised regarding the oncological efficiency of the procedure as initial reports documented a lower lymph node retrieval rate during the laparoscopic assisted gastrectomy (49, 50). These fears however seem to resolve as studies are steadily reporting similar results between laparoscopic and open surgery in regards to the lymph node retrieval efficiency (5, 36, 49–52). Recently, in the latest edition of the Japanese gastric cancer treatment guidelines 2014 (ver. 4) published by the Japanese Gastric Cancer Association, laparoscopic distal gastrectomy was upgraded from an investigational treatment to a valid option in general practice for clinical stage I cancer (49).

Optimally, the combination of laparoscopic surgery with sentinel node navigation in gastric cancer could further augment the advantages of each procedure with additional benefits for the patients (53). In general, the options for controlling the primary tumor are several depending on the certain oncological characteristics of the tumor itself (53). Endoscopic resection in the form of endoscopic sub-mucosal dissection can be applied to small mucosal cancers as long as the following conditions are fulfilled i.e., complete resection, tumor size < 2 cm in diameter, absence of a neoplastic ulcer, intestinal histologic type, pathologic T1a stage, negative lateral and vertical resection margins and absence of lympho-vascular invasion. In the case of larger and more aggressive cancer types other surgical options such as minimally invasive function-preserving gastrectomy or even conventional open gastrectomy should be considered (52).

## Is Sentinel Node Biopsy a Safe and Oncologically Efficient Approach?

Despite the recent obvious development on all aspects of sentinel node navigation surgery, there is still a notable controversy in the literature regarding the indications, the safety and the efficiency of the procedure oncologically-wise in gastric cancer. In general, numerous studies have already demonstrated pretty satisfactory results reporting a sentinel node detection rate of 90 to 100% and a metastasis detection sensitivity of up to 100% (Range: 85–100%) (36, 53–55).

There are two important prospective multicenter trials that aimed to test the sentinel node theory in early gastric cancer patients. The Japan Society of Sentinel Node Navigation Surgery study group conducted a multicenter prospective trial analyzing the validity of sentinel node navigation surgery utilizing a dual-tracer (radioactive colloid and isosulfan blue) (54). Three-hundred and 97 patients (397 pts.) gastric cancer patients of clinical cT1N0M0 or cT2N0M0 stage and a primary tumor of < 4 cm in diameter were included in the study. The reported sentinel node detection rate, the sensitivity and the overall accuracy of the technique were 97.5, 93.0, and 99%, respectively, results that lie within the previously reported data on the field (54, 56). The second study (JCOGO302) was performed by the JCOG aiming to evaluate the feasibility and the accuracy in diagnosis utilizing the sentinel node navigation surgery (35). Only patients with T1 cancers and tumor size of < 4 cm in diameter were enrolled in this study. According to the study design, the indocyanine green was used as a tracer which was injected at the sub—serosal layer around the primary tumor. Although the authors reported a totally acceptable detection rate of 97.8%, the rate of false-negative cases was unexpectedly high (46.4%) leading to a premature termination of the study.

In 2011, a meta-analysis of the existing feasibility studies was conducted aiming to examine the overall sensitivity of the sentinel node biopsy in gastric cancer patients (57). The authors reported a sentinel node identification rate and a sensitivity of 87.8 and 97.5%, respectively. In addition, the negative and positive predictive values were 91.8 and 38.0%, respectively. In the subgroup analysis, the sensitivity of sentinel node detection was shown to be depended on the number of the picked-up sentinel nodes. They concluded that sentinel node biopsy in gastric cancer is probably not clinically applicable for limited lymphadenectomy due to its unsatisfactory overall sensitivity and the significant heterogeneity observed between practicing surgeons. In order to improve the sensitivity, the authors proposed that a minimum of four sentinel nodes should be harvested and a sentinel basin dissection method should be applied (57).

Another meta-analysis published a year later by Wang et al. yielded quite different results i.e., sentinel node detection rate, sensitivity, negative predictive value and accuracy of 93.7, 76.9, 90.3, and 92.0%, respectively. The authors reported a significantly better sensitivity and detection rates when the dual tracer technique is used, when the tracer is injected in the sub-mucosa and when the picked up nodes are examined via immuno-histochemical staining (58). In general, studies from Eastern Asian institutions raise the accuracy of the procedure to as high as 98% in early stages (T1-T2 tumors) (25, 27) while the reported accuracy in Western institution studies was about 80%. The false negative sentinel lymph node rate ranges from 15 to 20% (59, 60). This notable variance in the results may be explained by the differences in the followed technique and the expertise of the involved surgeons on the procedure itself. The accurate detection is compromised in the case of skip metastases and this fact does seem to be influenced even by using the lymphatic basin dissection method. The incidence of skip metastasis, the major fear in this selective technique, was as high as 20% (61).

As quality of patients' life is increasingly becoming the crucial factor in evaluating the available treatment options, an approach of less invasive surgical procedures would be more often adapted in the future. In this direction, provided that sentinel node navigation surgery would achieve the desired accuracy levels, treatment individualization in the form of deviations from the established lymphadenectomy, currently the D2, could find their role in the gastric cancer surgical armamentarium. Certainly, the need for studies in the field of high level of evidence is obvious. The SENORITA trial, which compares the laparoscopic sentinel node biopsy or stomach preserving surgery (experimental arm) with the laparoscopy assisted gastrectomy with lymph node dissection (D1+ or more) (control arm) is ongoing and the first results are awaited by the end of 2020 (Trial registration number NCT01804998). Another, phase III trial is in progress as well (Trial registration number NCT01544413). The accumulation of the results of these clinical trials could indeed throw some additional light on the field and further clarify the role of the sentinel node navigation surgery in the gastric cancer treatment armamentarium.

However, there is still lot of things to be standardized in regards to the technique itself that would allow its widespread use. One of the most important is the part of the intra-operative pathology. The standard tactic today is to divide the frozen lymph node specimen into parts which are then in turn examined by using hematoxylin/eosin staining. The JCOG0302 trial revealed the inadequacy of the one plane frozen section examination to determine accurately the metastatic status of the retrieved node. Thus, multiple slice frozen section examination is mandatory in order to achieve high diagnostic accuracy (35). However, this approach is not free of limitations. Increasing the number of the samples to be examined, consumes time, increases the workload of surgeons and pathologists and ultimately increases the overall cost of the procedure. In order to reduce the number of false negative cases and overcome the limitation of the classic staining technique, the utilization of molecular-based diagnostic methods such as RT-PCR has been proposed (62, 63). Similarly, the RT-PCR procedure is also a time consuming method a fact that renders this sophisticated technique unpractical for the prompt diagnosis required during surgery (35, 36). A proper redesign of the technique with improvements in the result waiting time is obviously required (64).

A rapid and simple to use approach is the one-step nucleic acid amplification assay. This is an automated system that uses the reverse-transcription loop-mediated isothermal amplification method for gene amplification and might have the answers to the above mentioned limitations (65). Results are obtained usually within 30 min when a single node is to be examined. Optimally, this problem would require a solution in tune with the conditions within the operation room and the dye alone method as long it is improved in terms of accuracy to the levels of the dual tracer (dye and radioisotope) method would be ideal. Dye-method is simple and can be performed at any setting as no designated area or the use of sophisticated equipment is required for the injection and the interpretation of the results. Infra-red electronic endoscopy and indocyanine green fluorescence imaging are adjuncts that can increase the accuracy of the dye method but these detection systems require a darkroom for the visualization of the lymph node mapping. Recent studies suggested that the Hyper Eye Medical System, a system that can be used under day light of has the ability to visualize color and near-infrared rays at the same time (66, 67).

## Conclusions

In conclusion, sentinel node navigation surgery, validated appropriately, can probably play a significant, but well-defined, role in the definite treatment of gastric cancer creating, when appropriate, the conditions for targeted but oncologically effective lymphadenectomies. However, there are several problems on the technique itself and on the indications as well that require convincing answers such as easy to use and to visualize tracers in real operative time, brief and precise pathology and ultimately appropriate patient selection. In regards to tracers, combining tracers (dye plus isotope) seems to be the most effective and commonly followed approach due to the increased detection rates. Hematoxylin/eosin staining is still the mostly utilized method in regards to staining of the picked up nodes due to its overall advantages compared to the other more precise but the hard to use in everyday practice methods. The approach of lymphatic basin dissection seems promising because under certain circumstances could address and counterbalance existing limitations of the procedure in its current form. The lessons learned from the terminated study due to the unacceptable results, if appreciated appropriately, can turn from insurmountable obstacles to the stepping stone for actual progress on the field with direct clinical correspondence.

## Author Contributions

DS and KT made substantial contributions to the acquisition, the analysis, and the interpretation of the literature data. DS drafted the paper. DS and KT revised the paper critically for important intellectual content. Both authors gave their final approval of the final submitted manuscript.

### Conflict of Interest Statement

The authors declare that the research was conducted in the absence of any commercial or financial relationships that could be construed as a potential conflict of interest.
